# Postoperative Drainage Output as an Early Postoperative Marker Associated with Unplanned Revision After Soft Tissue Sarcoma Resection

**DOI:** 10.3390/diseases14080265

**Published:** 2026-07-23

**Authors:** Christian Spiegel, Riham Shanti, Friederike Weschenfelder, Michelle Mueller Spiegel, Maik Neumann, Mark Lenz, Wolfram Weschenfelder

**Affiliations:** 1 Department of Trauma, Hand and Reconstructive Surgery and Orthopaedics, University Hospital Jena, 07747 Jena, Germany; riham.shanti@uni-jena.de (R.S.); michelle.mueller@med.uni-jena.de (M.M.S.); maik.neumann@med.uni-jena.de (M.N.); mark.lenz@med.uni-jena.de (M.L.); wolfram.weschenfelder@med.uni-jena.de (W.W.); 2 Comprehensive Cancer Centre Central Germany, Campus Jena, 07747 Jena, Germany; 3 Department of Obstetrics, University Hospital Jena, 07747 Jena, Germany; friederike.weschenfelder@med.uni-jena.de

**Keywords:** soft tissue sarcoma, operation, revision, infection, drainage output

## Abstract

Background: Wound complications and unplanned revision surgery remain common after soft tissue sarcoma (STS) resection. Established risk models focus on patient- and tumour-related factors, while the role of operative and perioperative variables is less well defined. Methods: This retrospective single-centre study included 95 patients undergoing STS resection between 2021 and 2025. Associations with unplanned revision surgery were evaluated using univariate and exploratory multivariable logistic regression analyses. Receiver operating characteristic (ROC) analysis assessed the discriminatory performance of postoperative drainage output. Results: Unplanned revision surgery occurred in 23 patients (24%). On univariate analysis, several intraoperative and postoperative variables were associated with revision, whereas most patient- and tumour-related factors were not statistically significant. In multivariable analysis, intraoperative crystalloid administration was the only intraoperative factor associated with unplanned revision (OR 1.45 per 500 mL, 95% CI 1.05–2.02; *p* = 0.026). Among postoperative parameters, drainage output remained associated with revision (OR 1.45 per 100 mL, 95% CI 1.15–1.82; *p* = 0.005). In patients without planned secondary reconstruction, drainage output showed good exploratory discriminatory performance (AUC 0.927), with a cutoff of 925 mL providing high specificity (94.4%) with acceptable sensitivity (75%). Conclusions: In this cohort, unplanned revision was more frequently associated with markers of surgical complexity, including higher intraoperative crystalloid administration, and the early postoperative course, particularly drainage output. Drainage output appears to reflect an evolving complication rather than an independent predictor and may serve as an early clinical warning signal. Findings are hypothesis-generated and require external validation.

## 1. Introduction

Soft tissue sarcomas (STSs) require complex surgical management, often involving wide resections, large soft-tissue defects, and multimodal oncologic therapy. Despite advances in surgical technique and perioperative care, postoperative wound complications and surgical site infections (SSIs) remain common, with reported rates ranging from 15% to over 30%, particularly in tumours of the lower extremity and peripelvic region [1,2,3,4,5]. These complications are clinically relevant, as they frequently necessitate revision surgery, prolong hospitalisation, and may delay or preclude adjuvant treatment.

Numerous patient- and tumour-related risk factors for SSIs after STSs surgery have been identified, including advanced age, male sex, diabetes mellitus, smoking, obesity, impaired nutritional status, higher American Society of Anaesthesiologists (ASA) scores, large tumour size, deep or junctional location, lower extremity involvement, and neoadjuvant radiotherapy [1,3,6,7,8,9,10,11]. To facilitate risk stratification, several predictive models and nomograms have been developed based on patient- and tumour-related variables, while perioperative factors remain underrepresented [12,13].

In contrast, operation-specific and perioperative process variables have received comparatively little attention in the sarcoma literature. While operative time, blood loss, and transfusion requirements have been linked to increased infection risk, the broader influence of operative team composition, experience, and intraoperative management remains poorly defined [1,3,9,14]. Evidence from other surgical disciplines suggests that team-related and anaesthesia-related factors may significantly influence postoperative outcomes, yet comparable data for STS surgery are scarce [15,16,17,18].

Beyond risk factors, the clinical course and consequences of postoperative infection after STS resection are incompletely characterised. Although infection does not appear to adversely affect overall survival, it often results in unplanned revision surgery and may compromise timely oncologic treatment [19].

The primary aim was to identify factors associated with unplanned revision surgery following STS resection, with a focus on perioperative variables. Secondary analyses included postoperative infections and their clinical course.

## 2. Materials and Methods

### 2.1. Study Population

We retrospectively reviewed patients undergoing surgical treatment for soft tissue sarcomas at our institution between January 2021 and August 2025.

Of 144 eligible patients, 49 patients were excluded because of surgery for recurrent disease (*n* = 45), resection including bone (*n* = 1), or incomplete data (*n* = 3). The final cohort consisted of 95 patients. Ethical approval was granted (2025-4043-BO-D, 1 December 2025). Recurrent tumours were excluded because previous surgery and radiotherapy substantially alter tissue quality and wound healing, introducing a fundamentally different risk profile.

### 2.2. Perioperative Management

Postoperative wound drainage was applied routinely in all cases using at least one subfascial drain. Placement of a second drain was performed in cases involving multiple anatomical compartments or extensive resections at the discretion of the surgical team.Microbiological cultures were not obtained routinely but were collected in all cases requiring revision surgery. In these cases, at least three intraoperative tissue samples were obtained for microbiological analysis.

The primary endpoint was unplanned revision surgery, defined as any unplanned return to the operating theatre requiring a surgical procedure under general or regional anaesthesia (Clavien–Dindo grade IIIa or IIIb) for the management of postoperative wound-related complications. Total drainage output was calculated as the cumulative drainage volume recorded until drain removal.

### 2.3. Data Collection and Statistical Analysis

Clinical data, tumour characteristics, operative details, and imaging data were extracted from electronic records. Tumour size and Fujiwara classification were assessed on magnetic resonance imaging by an experienced musculoskeletal radiologist [20,21]. Intraoperative blood loss was estimated based on perioperative haemoglobin changes using the method described by Nadler and Good [22,23]. All procedures were jointly performed by the same two orthopaedic oncology surgeons (authors CS and WW) to minimise the potential influence of surgeon-related variability on revision rates.

Statistical analysis was performed using SPSS Statistics version 30.0 (IBM Corp., Armonk, NY, USA). All eligible patients were included; missing data were handled by case-wise exclusion without imputation. As most continuous variables were not normally distributed, non-parametric methods were applied. Continuous variables are presented as medians with interquartile ranges and were compared using the Mann–Whitney U test. Categorical variables were compared using Fisher’s exact test. Effect sizes were calculated using Cohen’s r and Cramer’s V, respectively.

Exploratory multivariable analysis was performed using binary logistic regression. Postoperative variables were not included to establish causal relationships but to characterise the early postoperative course. Given the limited number of events, multivariable analyses were explicitly defined as exploratory and restricted to a small number of clinically relevant variables to reduce the risk of model overfitting.

Total drainage output was further evaluated using receiver operating characteristic (ROC) curve analysis. The Youden index was used to inform exploratory assessment of potential cut-off values. For reasons of clarity and readability, baseline tables in the main manuscript were restricted to clinically relevant confounders and variables showing a potential association in univariate analysis (*p* < 0.10). Complete datasets are provided as supplementary material. All statistical tests were two-sided, and a *p* value <0.05 was considered statistically significant.

## 3. Results

### 3.1. Patient Characteristics

The final study cohort comprised 95 patients (median age 67 years, 54% male). Baseline characteristics are summarised in Table 1 and are restricted to clinically relevant confounders and variables showing a potential association with unplanned revision surgery (*p* < 0.10). The complete baseline dataset is provided in Supplementary Table S1.

### 3.2. Univariate Analysis

On univariate comparison, higher body weight was associated with an increased risk of unplanned revision. Larger tumour size was associated with unplanned revision (*p* = 0.016), while tumour location showed a borderline association (*p* = 0.05).

Several intraoperative factors were associated with unplanned revision, including vascular resection with reconstruction and higher intraoperative crystalloid administration.

Tumour location was associated with differences in postoperative drainage output. Median total drainage output was highest in lower extremity tumours (300 mL [IQR 200–1075]), followed by trunk lesions (450 mL [50–850]) and upper extremity tumours (100 mL [50–200]). Drainage output differed significantly between lower and upper extremity tumours (*p* < 0.001), while differences between trunk and other locations did not reach statistical significance. A borderline lower revision rate was also observed for upper limb tumours (*p* = 0.05). An exploratory analysis comparing medial thigh lesions with all other anatomical locations demonstrated significantly higher drainage volumes in medial thigh tumours (1300 mL [175–1700] vs. 200 mL [100–450], *p* = 0.037), although no association with revision surgery was observed (see Table 1 and Supplementary Table S1).

Postoperatively, patients undergoing unplanned revision had significantly longer intensive care unit/intermediate care unit (ICU/IMC) stays, prolonged drainage duration, markedly higher total drainage output, and substantially longer hospital stay.

### 3.3. Multivariable Analysis

In exploratory multivariable analysis, intraoperative crystalloid administration (OR 1.45 per 500 mL, 95% CI 1.05–2.02; *p* = 0.026) and preoperative weight (OR 1.03, 95% CI 1.001–1.058; *p* = 0.041) were independently associated with unplanned revision surgery (see Table 2). Among postoperative parameters, only total drainage output remained significantly associated with unplanned revision (OR 1.45 per 100 mL, 95% CI 1.15–1.82; *p* = 0.005), likely reflecting early manifestation of evolving wound complications rather than an independent causal factor.

### 3.4. Subgroup Analysis Excluding Planned Secondary Soft-Tissue Reconstruction

Univariate subgroup analysis excluding patients with planned secondary soft-tissue reconstruction is summarised in Supplementary Table S2. Several tumour- and procedure-related variables were associated with unplanned revision on univariate analysis.

In exploratory multivariable modelling, no pre- or intraoperative variables remained significantly associated with unplanned revision in this subgroup (Supplementary Table S3). In contrast, total drainage output remained independently associated with unplanned revision and was therefore retained in the main results (Table 3).

### 3.5. ROC Analysis

**Figure 1:**
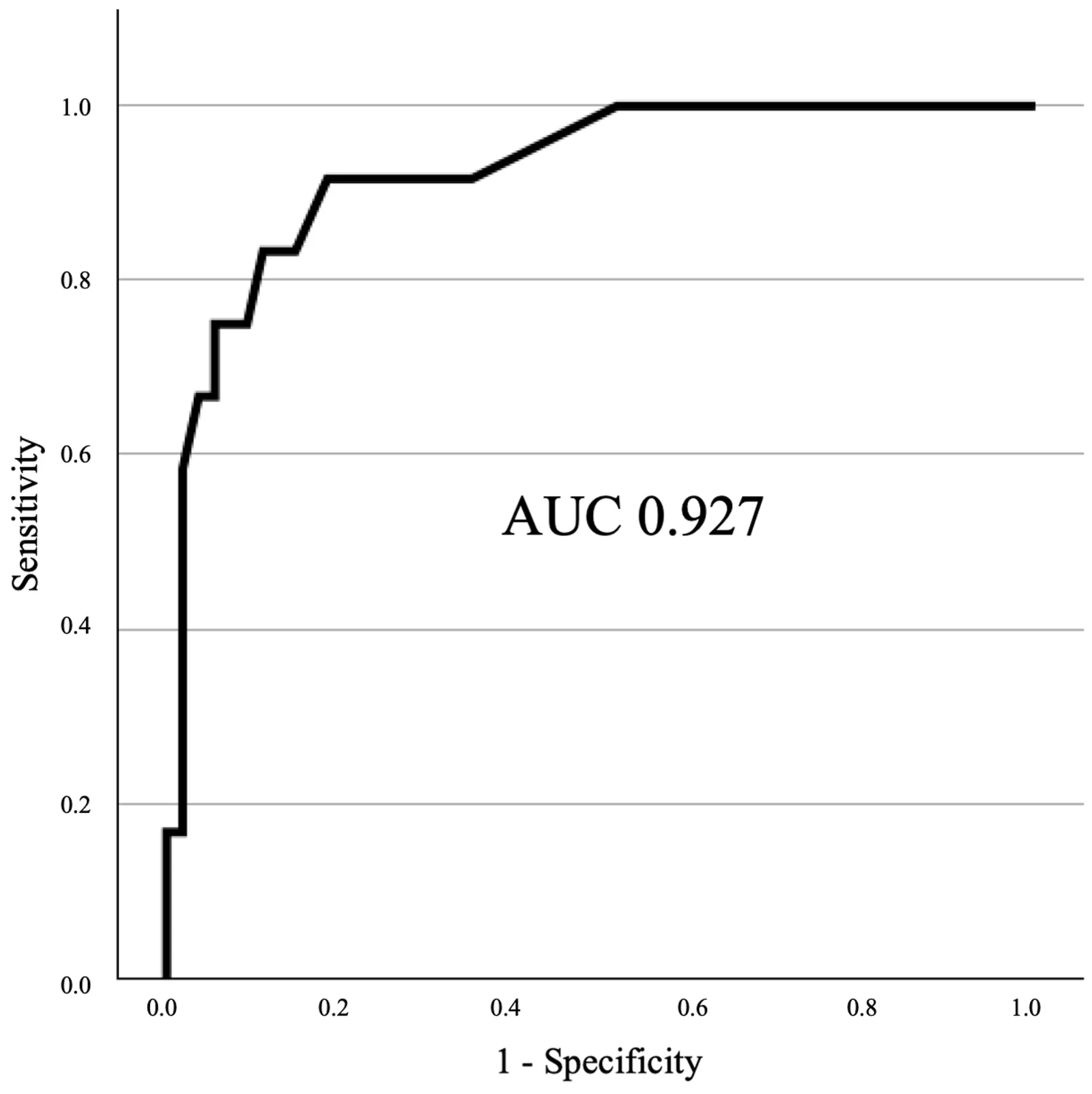
Receiver operating characteristics (ROC) curve of drainage output in the subgroup without planned secondary reconstruction.

Given the consistent independent association of total drainage output with unplanned revision surgery, a ROC analysis was performed in the subgroup excluding patients with planned secondary soft-tissue reconstruction. The area under the curve (AUC) was 0.927 (95% CI 0.849–1.000), indicating good exploratory discriminatory performance for identifying patients at increased risk during the early postoperative course (see Figure 1).

A cutoff value of 925 mL provided high specificity (94.4%) with acceptable sensitivity (75%) and was selected as an exploratory threshold for descriptive purposes. Further details of the ROC analysis and alternative cut-off considerations are provided in the Supplementary Material.

### 3.6. Descriptive Characteristics of Unplanned Revision Surgery

Descriptive characteristics of the 23 patients undergoing unplanned revision surgery are summarised in Table 4.

The median drain duration in patients undergoing unplanned revision was 5 days (IQR 3–7), whereas first revision surgery occurred on postoperative day 9 (IQR 7–22). Unplanned revision affected the course of adjuvant oncologic treatment in the majority of patients, leading to delayed therapy in 8 of 15 cases (54%) and cancellation in 2 of 15 cases (13%) among patients with evaluable infection outcome.

Microbiological cultures were positive in 14 patients (61%). Among these, Gram-positive organisms were isolated in five cases (36%), Gram-negative organisms in four cases (28%), and mixed flora in five cases (36%). Anaerobic bacteria were identified in three patients (21%). Staphylococcal species were the most frequently isolated pathogens (*n* = 7, 50%), followed by Escherichia coli (*n* = 4, 29%).

Infection was successfully controlled in 11 patients (92%), while persistent infection was observed in 1 patient (8%). During in-hospital stay, systemic disease progression occurred in four patients (33%), and one patient (7%) died due to progressive disease.

## 4. Discussion

This study suggests that, in this cohort, unplanned revision surgery after soft tissue sarcoma resection was more frequently observed in association with surgical complexity and the early postoperative course than with classical patient- or tumour-related risk factors (*n* = 95). However, established baseline risk factors did not reach statistical significance, likely reflecting limited statistical power rather than absence of effect.

In exploratory multivariable analysis, higher intraoperative crystalloid administration and increased postoperative drainage output were the variables most consistently associated with unplanned revision surgery. Tumour size and anatomical location showed associations in univariate analysis but did not remain independently significant after adjustment, likely reflecting limited sample size and overlap between markers of operative complexity. Importantly, these perioperative variables should be interpreted as surrogate markers of operative complexity and tissue disruption rather than independent causal determinants of wound complications.

Although tumour size and anatomical location were not independently associated with revision surgery in the multivariable model, both factors are likely to contribute to operative complexity. Larger tumours typically require wider resections with increased dead space formation, while lower extremity procedures—particularly around the proximal and medial thigh—are associated with extensive soft tissue mobilisation and potential lymphatic disruption. These mechanisms may influence postoperative drainage indirectly through surgical extent rather than acting as independent determinants of revision. Direct measures of lymphatic injury or local fluid dynamics were not available, and mechanistic interpretation therefore remains speculative.

Patient body weight was also independently associated with unplanned revision surgery in the exploratory multivariable analysis. Similar to intraoperative crystalloid administration, this finding should be interpreted cautiously. Higher body weight may reflect increased subcutaneous tissue volume and larger surgical wound surfaces, potentially contributing to greater dead space formation and postoperative exudate. However, given the interrelationship between body habitus, operative extent, and tissue handling, this variable is more likely to represent a surrogate of operative complexity rather than an independent causal determinant [7,24].

Although vascular reconstruction was excluded from multivariable modelling due to low case numbers, all affected patients required unplanned revision. This observation aligns with existing literature reporting high complication rates following limb salvage surgery with vascular reconstruction and highlights this subgroup as particularly vulnerable, despite the surgical necessity of such procedures [25].

While radiotherapy is a well-established risk factor for postoperative complications, no statistically significant association was observed in the present cohort, likely due to limited sample size [26]. Accordingly, the absence of statistical significance should not be considered evidence of no clinical effect.

Higher intraoperative crystalloid administration was independently associated with unplanned revision surgery in the exploratory multivariable model. However, fluid administration itself is unlikely to exert a direct biological effect on wound healing. Rather, increased crystalloid requirements probably reflect more extensive procedures, larger wound cavities, greater tissue manipulation, and increased physiological turnover during surgery.

A central finding of this study is the consistent association between postoperative drainage output and unplanned revision surgery. Postoperative drainage output remained the only parameter consistently associated with unplanned revision after adjustment and subgroup analysis, and demonstrated good exploratory discriminatory performance in ROC analysis. An internally derived threshold of approximately 925 mL was associated with high specificity within this cohort. However, given the limited sample size and exploratory nature of the analysis, this threshold should not be interpreted as a clinically validated decision limit but rather as a hypothesis-generating observation requiring external validation. Because cumulative drainage output was analysed until drain removal, measurements may have included values obtained after early clinical deterioration or after the decision to perform revision. Accordingly, drainage output should be interpreted as a marker of the evolving postoperative course rather than a predictor necessarily preceding clinical deterioration.

From a pathophysiological perspective, increased postoperative drainage output likely reflects underlying mechanisms such as persistent dead space, inadequate hemostasis, lymphatic leakage, or early inflammatory or infectious exudate. Importantly, postoperative drainage may include expected baseline serous exudate following wide oncologic resection, which could not be separately quantified in this study. These mechanisms are closely related to surgical extent, anatomical location, and local tissue conditions. In particular, anatomical regions with a higher lymphatic burden, such as the proximal and medial lower extremity, may be more prone to prolonged drainage due to lymphatic disruption. However, the exact composition of postoperative wound drainage was not analysed in the present study, and lymphatic leakage therefore remains a plausible but speculative mechanistic explanation. Persistent postoperative fluid leakage and seroma formation remain important challenges after tumour resection across various surgical subspecialties, underscoring the need for further research into preventive strategies [27,28,29,30].

The observed variation in drainage output across anatomical locations in the present cohort supports this interpretation, as larger compartments and more extensive resections—particularly in the lower extremity—were associated with higher drainage volumes. These findings should be interpreted as exploratory and descriptive rather than inferential regarding causal pathways. An additional exploratory analysis of medial thigh tumours demonstrated significantly greater drainage volumes compared with other anatomical locations, although no statistically significant association with revision surgery itself was observed. This observation supports the concept that anatomical factors may influence postoperative wound secretion, while also illustrating that increased drainage alone is unlikely to fully explain the occurrence of revision surgery.

Importantly, postoperative variables such as drainage output, ICU stay, or length of hospitalisation must be interpreted in the context of the evolving postoperative course. These parameters represent early manifestations of postoperative complications rather than independent predictors. Accordingly, drainage output should be considered a surveillance marker prompting closer clinical monitoring rather than a standalone indication for surgical intervention.

### Strengths and Limitations

Strengths of this study include detailed perioperative data and a consistent surgical approach. Limitations include the retrospective design, single-centre setting, and limited number of events, restricting causal inference. The limited number of revision events restricts the robustness of multivariable modelling. Given the small number of events, multivariable analyses are susceptible to overfitting and model instability. In addition, several variables likely reflect overlapping aspects of surgical complexity, increasing the risk of collinearity.

Overall, multivariable findings should be interpreted as hypothesis-generating. The inclusion of postoperative variables further limits causal interpretation. The proposed drainage cutoff requires external validation. Although operative duration and tumour size were available, other important determinants of postoperative drainage, including dead-space volume, number of drains, extent of soft-tissue mobilisation, reconstructive complexity and direct assessment of lymphatic injury, were unavailable. Residual confounding by surgical complexity therefore cannot be excluded. Because cumulative drainage output was analysed until drain removal, temporal separation between increasing drainage and clinical recognition of complications cannot be guaranteed.

Furthermore, the exact nature of postoperative drainage output (e.g., lymphatic, serous, or infectious) could not be differentiated in this study, limiting mechanistic interpretation.

## 5. Conclusions

In this cohort, unplanned revision surgery after STS resection was associated with surrogate markers of operative complexity and the early postoperative course rather than baseline patient or tumour characteristics. These associations likely reflect combined effects of surgical extent, tissue disruption, and postoperative fluid dynamics rather than discrete causal mechanisms. Increased postoperative drainage output appears to reflect evolving wound complications rather than an independent causal risk factor and may therefore serve as an early clinical surveillance marker. It should not be interpreted as a standalone decision threshold for reintervention. These findings should be regarded as hypothesis-generating and require validation in larger, preferably prospective, multicentre cohorts before clinical implementation.

## Figures and Tables

**Table 1:** Baseline characteristics of patients with and without unplanned revision surgery in the overall cohort, restricted to clinically relevant confounders and variables with *p* < 0.10 in univariate analysis. The complete baseline dataset is provided in Supplementary Table S1.

	No Unplanned Revision (*n* = 72)	Unplanned Revision (*n* = 23)	*p*-Value	Effect Size
Patient Factors				
Female	34	10	0.81	0.03
Male	38	13		
Age in years	68 (55–81)	67 (52–74)	0.152	0.15
Weight in kg	78.0 (67.8–88.0)	89.0 (77.0–100.0)	0.044 *	0.21
BMI in kg/m^2^	26.5 (24.0–29.7)	27.8 (25.6–34.4)	0.099	0.17
Peripheral arterial disease	0	2	0.057	0.26
No peripheral arterial disease	72	21		
Tumour Factors				
Maximal tumour size in cm	8.1 (5.2–12.7)	12.5 (9.2–17.2)	0.016 *	0.25
Pleomorphic sarcoma	24	6	0.121	0.28
Liposarcoma (except ALT)	6	3		
Myxofibrosarcoma	9	8		
Other high-grade sarcoma	22	5		
Low-grade sarcoma	11	1		
Lower limb	36	17	0.050 *	0.24
Upper limb	24	2		
Trunk	12	4		
Medial thigh compartment	5	4	0.21	0.15
Other localisation	67	19		
No neoadjuvant treatment	43	10	0.134	0.25
Radiation	11	4		
Radiochemotherapy	13	9		
Chemotherapy	5	0		
Intraoperative Factors				
Operative time in min	75 (52–107)	102 (57–148)	0.069	0.19
Vascular reconstruction	0	3	0.013 *	0.32
No vascular reconstruction	72	20		
Necessity for secondary flap	18	11	0.067	0.21
Primary closure	54	12		
Intraoperative crystalloids in L	1.0 (0.6–2.0)	1.5 (1.0–2.0)	0.020 *	0.24
Postoperative Factors				
Intensive/intermediate care days	0(0–0)	0(0–1)	0.002 *	0.32
Drainage duration in days	3 (2–4)	5 (4–7)	0.006 *	0.33
Drainage output in mL	200 (63–338)	1425 (775–1750)	<0.001 *	0.56
Outcome				
Length of hospital stay in days	12 (8–18)	28 (16–41)	<0.001 *	0.52

Variables were selected a priori based on clinical relevance and exploratory univariate association (*p* < 0.10). ALT—atypical lipomatous tumour, ASA—American Society of Anaesthesiologists; BMI—Body mass index metric data: median with Inter-Quartile-Range, Mann–Whitney U Test, Cohen’s r for effect size nominal data: number (%), Fisher’s exact test, Cramer’s V for effect size. * indicates statistical significance with *p* < 0.05.

**Table 2:** Exploratory multivariable analysis of risk factors for unplanned revision surgery in the overall cohort.

	*p*-Value	OR	CI
Pre- And Perioperative Model Variables			
Weight in kg	0.041 *	1.03	1.001–1.058
Maximal tumour size in cm	0.60	1.02	0.95–1.10
Lower limb	0.147		
Upper limb	0.051	0.18	0.032–1.006
Trunk	0.84	0.86	0.22–3.40
Intraoperative crystalloids in 500 mL	0.026 *	1.45	1.05–2.02
Postoperative Model Variables			
Intensive/intermediate care days	0.155	2.77	0.68–11.29
Drainage duration in days	0.68	0.86	0.42–1.77
Drainage output in 100 mL	0.005 *	1.45	1.15–1.82

Binary logistic regression models, OR—Odds Ratio, CI—Confidence Interval. Due to the limited number of events, variables with very low prevalence were excluded from multivariable modelling. Reference categories for categorical data: Lower limb. * indicating statistical significance with *p* < 0.05.

**Table 3:** Exploratory multivariable analysis of postoperative variables associated with unplanned revision surgery in patients without planned secondary soft-tissue reconstruction.

	*p*-Value	OR	CI
Intensive/intermediate care days	0.170	2.67	0.66–10.83
Drainage duration in days	0.64	0.84	0.40–1.76
Drainage output in 100 mL	0.002 *	1.45	1.15–1.82

Binary logistic regression model, OR—Odds Ratio, CI—Confidence Interval.* indicating statistical significance with *p* < 0.05.

**Table 4:** Descriptive characteristics of patients undergoing unplanned revision surgery (*n* = 23).

Variable	Revision Cohort (*n* = 23)
Main indication for first revision	
- Seroma	10 (44)
- Surgical site infection	8 (35)
- Persistent wound leakage	4 (17)
- Systemic infection	1 (4)
Median postoperative day of first revision	9 (7–22)
Median number of revision procedures	3 (2–4)
Positive microbiology	14 (61)
Secondary plastic reconstruction	15 (65)
Flap reconstruction	10 (45)
Split skin graft	5 (23)

Nominal data: number (%); metric data: median with Inter-Quartile-Range.

## Data Availability

The data presented in this study are not publicly available but available on request from the corresponding author. The data are not publicly available due to privacy and ethical restrictions.

## Supplementary Materials

The following supporting information can be downloaded at: https://www.mdpi.com/article/10.3390/diseases14080265/s1, Supplementary Table S1. Complete baseline characteristics of patients with and without unplanned revision surgery in the overall cohort. Supplementary Table S2. Univariate subgroup analysis excluding patients with planned secondary soft-tissue reconstruction. Supplementary Table S3. Exploratory multivariable analysis of pre- and intraoperative variables associated with unplanned revision surgery in patients without planned secondary soft-tissue reconstruction. Supplementary Results S1. Detailed ROC analysis of total drainage output.

## Abbreviations

The following abbreviations are used in this manuscript:

AUC	Area under the curve
ASA	American Society of Anaesthesiologists
BMI	Body mass index
CI	Confidence interval
ICU/IMC	Intensive care unit/Intermediate care unit
OR	Odds ratio
ROC	Receiver operating characteristic
SSI	Surgical site infection
STS	Soft tissue sarcoma
