# Supplementary material for: Postoperative Drainage Output as an Early Postoperative Marker Associated with Unplanned Revision After Soft Tissue Sarcoma Resection
Source: Diseases. 2026 Jul 23;14(8):265. doi: 10.3390/diseases14080265 (PMC13512249; doi:10.3390/diseases14080265)
Supplement: Supplementary file 1 [file diseases-14-00265-s001.zip › diseases-4311667-supplementary.pdf]

Supplementary Table S1. Complete baseline characteristics of patients with and without unplanned revision surgery in the overall cohort.

	No unplanned revision (n=82)	Unplanned revision (n=23)	p-value	effect size
PATIENT FACTORS				
Female<br>Male	34<br>38	10<br>13	0.81	0.03
Age in years	68<br>(55 – 81)	67<br>(52 – 74)	0.152	0.15
Size in cm	170<br>(164 – 175)	172<br>(164 – 178)	0.62	0.05
Weight in kg	78.0<br>(67.8 – 88.0)	89.0<br>(77.0 – 100.0)	0.044*	0.21
BMI in kg/m^2	26.5<br>(24.0 – 29.7)	27.8<br>(25.6 – 34.4)	0.099	0.17
Smoking<br>No smoking	17<br>55	9<br>14	0.181	0.15
Diabetes<br>No Diabetes	13<br>59	4<br>19	1.00	0.01
Peripheral arterial disease<br>No peripheral arterial disease	0<br>72	2<br>21	0.057	0.26
Immunosuppression<br>No Immunosuppression	1<br>71	1<br>22	0.43	0.09
No anticoagulation<br>Therapeutic anticoagulation<br>Platelet inhibitors<br>Combination of above	19<br>36<br>5<br>12	3<br>12<br>0<br>7	0.24	0.22
Albumin in mmol/L	41<br>(39 – 44)	43<br>(40 – 45)	0.41	0.09
Creatinine in mmol/L	81<br>(68 – 95)	77<br>(66 – 89)	0.45	0.08
Preoperative haemoglobin in mmol/L	8.0<br>(7.0 – 9.1)	7.7<br>(7.2 – 9.1)	0.98	0.00
Depression<br>No Depression	1<br>71	2<br>21	0.144	0.18
ASA-Score I<br>ASA-Score II<br>ASA-Score III	3<br>39<br>29	1<br>8<br>14	0.176	0.18
TUMOUR FACTORS				
Palliative Treatment Intent<br>Curative Treatment Intent	6<br>63	1<br>21	1.00	0.07
Maximal tumour size in cm	8.1<br>(5.2 – 12.7)	12.5<br>(9.2 – 17.2)	0.016*	0.25
Pleomorphic sarcoma<br>Liposarcoma (except ALT)<br>Myxofibrosarcoma<br>Other high-grade sarcoma<br>Low-grade sarcoma	24<br>6<br>9<br>22<br>11	6<br>3<br>8<br>5<br>1	0.121	0.28
Lower limb<br>Upper limb<br>Trunk	36<br>24<br>12	17<br>2<br>4	0.050*	0.24
Thigh – extensors	9	7	0.60	0.24

Thigh – flexors<br>Thigh – adductors<br>Thigh – other<br>Lower limb – other	10<br>5<br>4<br>8	3<br>4<br>1<br>2		
Medial thigh compartment<br>Other localisation	5<br>67	4<br>19	0.21	0.15
Superficial<br>Deep	8<br>64	1<br>22	0.45	0.10
Fujiwara type I<br>Fujiwara type II<br>Fujiwara type III<br>Fujiwara type IV	53<br>5<br>10<br>4	14<br>2<br>3<br>4	0.32	0.19
Previous whoops incision<br>No previous whoops incision	3<br>69	1<br>22	1.00	0.00
No neoadjuvant treatment<br>Radiation<br>Radiochemotherapy<br>Chemotherapy	43<br>11<br>13<br>5	10<br>4<br>9<br>0	0.134	0.25
No neoadjuvant treatment<br>Any neoadjuvant treatment	43<br>29	10<br>13	0.23	0.14
INTRAOPERATIVE FACTORS				
1st operation of day<br>2nd operation of day<br>3rd operation of day<br>4th operation of day	32<br>31<br>7<br>2	15<br>7<br>1<br>0	0.42	0.19
Orthopaedic theatre<br>Other theatre	54<br>18	15<br>8	0.42	0.09
Operative time in min	75<br>(52 – 107)	102<br>(57 – 148)	0.069	0.19
Vascular reconstruction<br>No vascular reconstruction	0<br>72	3<br>20	0.013*	0.32
Intraoperative blood loss<br>in L	0.6<br>(0.3 – 0.8)	0.6<br>(0.4 – 1.1)	0.34	0.10
Orthopaedic scrub nurse<br>Other scrub nurse	46<br>26	13<br>10	0.62	0.07
Orthopaedic scout nurse<br>Other scout nurse	25<br>47	9<br>14	0.80	0.04
1st anaesthetist experience<br>- registrar year 1<br>- registrar year 2-3<br>- registrar year 4-5<br>- specialist<br>- consultant	 35<br>17<br>10<br>8<br>2	 13<br>4<br>2<br>3<br>1	0.87	0.11
2nd anaesthetist experience<br>- registrar year 2-3<br>- registrar year 4-5<br>- specialist<br>- consultant	 1<br>0<br>16<br>53	 0<br>1<br>9<br>13	0.114	0.25
No perioperative antibiosis<br>Cefazolin<br>Clindamycin	2<br>69<br>1	0<br>22<br>1	0.68	0.12
Necessity for secondary flap	18	11	0.067	0.21

Primary closure	54	12		
Limb salvage surgery<br>Amputation	61<br>11	21<br>2	0.73	0.08
Intraoperative crystalloids in L	1.0<br>(0.6 – 2.0)	1.5<br>(1.0 – 2.0)	0.020*	0.24
POSTOPERATIVE FACTORS				
Intensive/intermediate care days	0<br>(0 – 0)	0<br>(0 – 1)	0.002*	0.32
Drainage duration in days	3<br>(2 – 4)	5<br>(4 – 7)	0.006*	0.33
Drainage output in mL	200<br>(63 – 338)	1425<br>(775 – 1750)	<0.001*	0.56
OUTCOME				
Length of hospital stay in days	12<br>(8 – 18)	28<br>(16 – 41)	<0.001*	0.52

ALT – atypical lipomatous tumour, ASA – American Society of Anaesthesiologists; BMI – Body mass
metric data: median with Inter-Quartile-Range, Mann-Whitney-U-Test, Cohen's r for effect size
nominal data: number of cases and percentage of column, Fisher's exact test, Cramer's V for effect size
** indicating statistical significance with p < 0.05*

Supplementary Table S2. Univariate subgroup analysis excluding patients with planned secondary soft-tissue reconstruction.

	No revision (n=54)	Revision (n=12)	p-value	effect size
PATIENT FACTORS				
Female<br>Male	24<br>30	6<br>6	0.76	0.04
Age in years	71<br>(56 – 81)	66<br>(53 – 77)	0.22	0.15
Size in cm	170<br>(164 – 175)	165<br>(161 – 181)	0.46	0.09
Weight in kg	79<br>(69 – 89)	80<br>(67 – 90)	0.58	0.07
BMI in kg/m^2	26.5<br>(23.5 – 29.8)	26.8<br>(24.0 – 32.9)	0.45	0.09
Smoking<br>No smoking	9<br>45	3<br>9	0.68	0.08
Diabetes<br>No Diabetes	9<br>45	3<br>9	0.68	0.08
Peripheral artery disease<br>No peripheral artery disease	0<br>54	0<br>12	-	-
Immunosuppression<br>No Immunosuppression	1<br>53	1<br>11	0.33	0.15
No anticoagulation<br>Therapeutic anticoagulation<br>Platelet inhibitors<br>Combination of above	17<br>24<br>5<br>8	1<br>6<br>0<br>4	0.186	0.28
Albumin in mmol/L	41<br>(39 – 44)	42<br>(31 – 46)	0.74	0.04
Creatinine in mmol/L	80	73	0.51	0.08

	(67 – 91)	(66 – 89)		
Preoperative haemoglobin<br>in mmol/L	8.0<br>(7.0 – 9.1)	7.6<br>(6.8 – 8.4)	0.26	0.14
Depression<br>No Depression	0<br>54	2<br>12	0.031*	0.38
ASA-Score I<br>ASA-Score II<br>ASA-Score III	2<br>30<br>21	1<br>4<br>7	0.22	0.19
TUMOUR FACTORS				
Palliative Treatment Intent<br>Curative Treatment Intent	5<br>47	1<br>11	1.00	0.02
Maximal tumour size in cm	8.9<br>(5.8 – 14.1)	12.7<br>(12.1 – 19.8)	0.005*	0.34
Pleomorphic sarcoma<br>Liposarcoma (except ALT)<br>Myxofibrosarcoma<br>Other high-grade sarcoma<br>Low-grade sarcoma	18<br>6<br>8<br>14<br>8	3<br>2<br>4<br>2<br>1	0.60	0.21
Lower limb<br>Upper limb<br>Trunk	24<br>20<br>10	10<br>1<br>1	0.052	0.30
Thigh – extensors<br>Thigh – flexors<br>Thigh – adductors<br>Thigh – other<br>Lower limb – other	7<br>6<br>5<br>2<br>4	4<br>0<br>4<br>1<br>0	0.25	0.41
Medial thigh compartment<br>Other localisation	5<br>49	4<br>8	0.050*	0.27
Superficial<br>Deep	2<br>52	0<br>12	1.00	0.08
Fujiwara I<br>Fujiwara II<br>Fujiwara III<br>Fujiwara IV	37<br>4<br>9<br>4	4<br>1<br>3<br>4	0.035*	0.34
Previous whoops incision<br>No previous whoops incision	2<br>52	0<br>12	1.00	0.08
No neoadjuvant treatment<br>Radiation<br>Radiochemotherapy<br>Chemotherapy	31<br>7<br>12<br>4	3<br>3<br>6<br>0	0.076	0.32
No neoadjuvant treatment<br>Any neoadjuvant treatment	31<br>23	3<br>9	0.058	0.25
INTRAOPERATIVE FACTORS				
1st operation of day<br>2nd operation of day<br>3rd operation of day<br>4th operation of day	27<br>20<br>6<br>1	9<br>3<br>0<br>0	0.43	0.22
Orthopaedic theatre<br>Other theatre	41<br>13	9<br>3	1.00	0.01
Operative time in min	77<br>(52 – 116)	143<br>(87 – 339)	0.003*	0.37
Vascular reconstruction	0	3	0.005*	0.46

No vascular reconstruction	54	9		
Intraoperative Blood loss in L	0.5 (0.3 – 0.8)	0.5 (0.4 – 0.7)	0.77	0.04
Orthopaedic scrub nurse<br>Other scrub nurse	33<br>21	6<br>6	0.53	0.09
Orthopaedic scout nurse<br>Other scout nurse	18<br>36	3<br>9	0.74	0.07
1st anaesthetist experience<br>- registrar year 1<br>- registrar year 2-3<br>- registrar year 4-5<br>- specialist<br>- consultant	 25<br>15<br>7<br>6<br>1	 8<br>2<br>0<br>2<br>0	0.56	0.22
2nd anaesthetist experience<br>- registrar year 2-3<br>- registrar year 4-5<br>- specialist<br>- consultant	 1<br>0<br>13<br>39	 0<br>1<br>5<br>6	0.132	0.32
No perioperative antibiosis<br>Cefazolin<br>Clindamycin	2<br>51<br>1	0<br>12<br>0	1.00	0.10
Limb salvage surgery<br>Amputation	44<br>10	10<br>2	1.00	0.02
Intraoperative crystalloids in L	1.0 (0.5 – 2.0)	2.0 (1.0 – 3.8)	0.047*	0.24
POSTOPERATIVE FACTORS				
Intensive/intermediate care days	0 (0 – 0)	0 (0 – 1)	0.004*	0.36
Drainage duration in days	3 (2 – 4)	5 (4 – 7)	<0.001*	0.42
Drainage output in mL	200 (50 – 300)	1425 (775 – 1750)	<0.001*	0.57
OUTCOME				
Length of hospital stay in days	10 (7 – 14)	26 (14 – 51)	<0.001*	0.48

ALT – atypical lipomatous tumour, ASA – American Society of Anaesthesiologists; BMI – Body mass
metric data: median with Inter-Quartile-Range, Mann-Whitney-U-Test, Cohen's r for effect size
nominal data: number of cases and percentage of column, Fisher's exact test, Cramer's V for effect size
** indicating statistical significance with $p < 0.05$*

Supplementary Table S3. Exploratory multivariable analysis of pre- and intraoperative variables associated with unplanned revision surgery in patients without planned secondary soft-tissue reconstruction.

	p-value	OR	CI
Maximal tumour size in cm	0.43	1.04	0.94 – 1.16
Medial thigh compartment<br>Other localisation	0.55	2.27	0.16 – 31.94
Fujiwara Type I<br>Fujiwara Type II<br>Fujiwara Type III<br>Fujiwara Type IV	0.49<br>0.47<br>0.36<br>0.38	 0.30<br>0.20<br>2.89	 0.01 – 7.73<br>0.01 – 6.50<br>0.27 – 30.93
Operative time in min	0.068	1.012	0.999 – 1.026
Intraoperative crystalloids in L	0.55	1.23	0.62 – 2.55

Binary logistic regression model, OR – Odds Ratio, CI – Confidence Interval, n/a – not applicable (due to small number), Due to the limited number of events, variables with very low prevalence were excluded from multivariable modelling.
Reference categories for categorial data: Fujiwara Type I
** indicating statistical significance with $p < 0.05$*

Supplementary Results S1. Detailed ROC analysis of total drainage output

Given the strong and consistent association of total drainage output with unplanned revision surgery in both the overall cohort and subgroup analyses, a detailed receiver operating characteristic (ROC) analysis was performed to further explore its discriminatory performance.

In the subgroup excluding patients with planned secondary soft-tissue reconstruction, the ROC curve yielded an area under the curve (AUC) of 0.927 (95% CI 0.849–1.000), indicating excellent discrimination. The maximum Youden index of 0.731 was observed at a cut-off value of 425 mL, corresponding to a sensitivity of 91.7% and a specificity of 81.5%. Application of this threshold would have resulted in 44 true negatives, 10 false positives, 1 false negative, and 11 true positives.

However, given the relatively high proportion of false-positive classifications (10/66) and the resulting risk of unnecessary clinical interventions, an alternative cut-off prioritising high specificity was selected. Using a cut-off of 925 mL (Youden index 0.694), sensitivity was 75% and specificity 94.4%, yielding 51 true negatives, 3 false positives, 3 false negatives, and 9 true positives.

From a clinical perspective, this higher threshold was considered more appropriate for identifying patients at genuinely increased risk of unplanned revision surgery while minimising overtreatment.
